# Improvement of Cancer Prevention and Control: Reflection on the Role of Emerging Information Technologies

**DOI:** 10.2196/50000

**Published:** 2024-02-27

**Authors:** Pei Dong, Ayan Mao, Wuqi Qiu, Guanglin Li

**Affiliations:** 1 Department of Public Health Strategy Research Institute of Medical Information Chinese Academy of Medical Sciences and Peking Union Medical College Beijing China; 2 Chinese Preventive Medicine Association Beijing China

**Keywords:** emerging information technologies, cancer, prevention and control

## Abstract

Cancer has become an important public health problem affecting the health of Chinese residents, as well as residents all over the world. With the improvement of cancer prevention and treatment, the growth of the mortality rate of cancers has slowed down gradually, but the incidence rate is still increasing rapidly, and cancers still impose heavy disease and economic burdens. Cancer screening and early cancer diagnosis and treatment are important ways to reduce the burden of cancer-related diseases. At present, various projects for early cancer diagnosis and treatment have been implemented in China. With the expansion of the coverage of these projects, the problems related to project implementation, operation, and management have emerged gradually. In recent years, emerging information technologies have been applied in the field of health and have facilitated health management and clinical decision-making. Meanwhile, China announced multiple policies to encourage and promote the application of information technologies in the field of health. Therefore, combined with the analysis of major problems in cancer prevention and control projects, this paper probes into how to apply information technologies such as biological information mining, artificial intelligence, and electronic information collection technology to various stages of cancer prevention and control. Information technologies realize the integrated management of prevention and control processes, for example, mobilization and preliminary identification, high-risk assessment, clinical screening, clinical diagnosis and treatment, tracking and follow-up, and biological sample management of high-risk groups, and promote the efficient implementation of cancer prevention and control projects in China.

## Background

**Figure 1:**
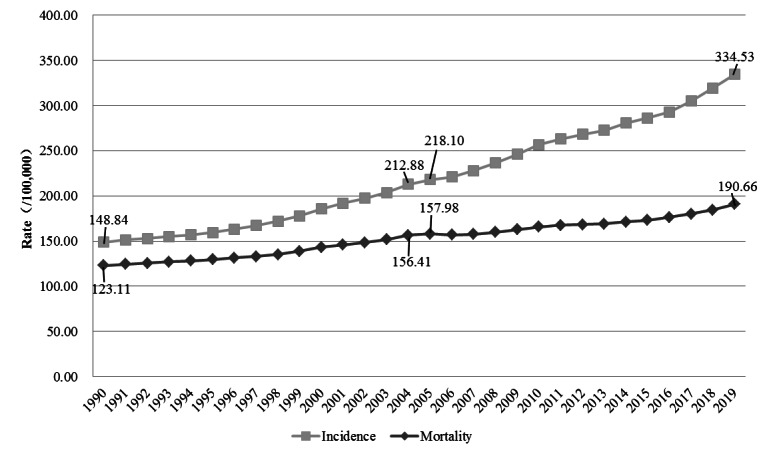
Incidence and mortality of all-sex cancers in China from 1990 to 2019.

Cancer has become the leading cause of death in many countries in the world [1]. It is an important public health problem affecting the health of people all over the world, including Chinese people. In recent years, with the improvement of cancer prevention and treatment, the growth of the mortality rate of cancers in China slowed down gradually. The all-sex mortality rate of cancers increased from 123.11 per 100,000 persons in 1990 to 156.41 per 100,000 persons in 2004 [2] (with an average growth rate of about 2.57%) and increased from 157.98 per 100,000 persons in 2005 to 190.66 per 100,000 persons in 2019 [2] (with an average growth rate of about 1.81%). However, the incidence rate is still increasing rapidly. The all-sex crude incidence of cancers increased from 148.84 per 100,000 persons in 1990 to 212.88 per 100,000 persons in 2004 [2] (with an average growth rate of about 3.45%) and increased from 218.10 per 100,000 persons in 2005 to 334.53 per 100,000 persons in 2019 [2] (with an average growth rate of about 3.56%; Figure 1). In China, cancers still impose a heavy disease burden. The rate of standardized disability-adjusted life years for cancers in China showed a downward trend during the years from 1990 to 2019, but it is still higher than the world average level [3]. The economic burden caused by cancers in China should not be ignored. Some studies have pointed out that the direct economic burden caused by cancers in China in 2003 and 2010 was CN ¥32.63 billion (US $5.28 billion) and CN ¥100.74 billion (US $163.30 billion), respectively [4], accounting for 4.96% and 5.04% of the total health expenditure in that year respectively.

A large number of studies have confirmed that cancer screening and early cancer diagnosis and treatment are important ways to reduce the burden of cancer-related diseases [5]. To increase the rate of early cancer diagnosis and treatment, a variety of early cancer diagnosis and treatment projects have been implemented since 2005 [6], which have played a positive role in reducing the health hazards of cancers. With the expansion of the coverage of the projects; the increase in the number of participants; and the accumulation of massive evaluation, screening, and complicated data, higher requirements have been raised for the implementation and management of cancer prevention and control screening projects. In recent years, China announced multiple policies to encourage and promote the application of information technologies to the field of health [7-9]. The *National Health Plan for the 14th Five-Year Plan Period* requires that emerging information technologies, for example, artificial intelligence (AI) and big data technologies, should be popularized and applied to provide intelligent medical services and chronic disease screening services [10]. At present, most of the projects in China’s cancer prevention and control working modes are still based on traditional methods (eg, collecting information by paper questionnaires and manual entry), while emerging information technologies have been more maturely applied to similar scenarios in the health field. We, therefore, aimed to reflect on the combination of emerging information technologies and cancer prevention and control projects in China, build a bridge that may persuade China’s tumor prevention and control management agencies to make full use of information technology, promote the efficiency of the management, and benefit more residents and medical staff.

## Main Cancer Prevention and Control Projects and Their Working Modes in China

### Cancer Prevention and Control Projects in China

China’s current cancer prevention and control programs are roughly divided into 3 categories, that is, national-level public welfare projects, local-level public welfare projects, and public welfare projects implemented by charity organizations.

As major specific public health services in the country, national-level cancer screening projects include *Cancer Screening Program in Rural Areas* (2005), *Cancer Screening Program in Huaihe River Area* (2007), *Cervical Cancer and Breast Cancer Screening Program for Women* (2009; included in the basic public health service in 2019), and *Cancer Screening Program in Urban Areas* (2012). These cover such high-incidence cancers as lung cancer, stomach cancer, esophageal cancer, colorectal cancer, liver cancer, breast cancer, and cervical cancer. These projects enable related people, for example, high-incidence populations and special populations in rural, urban, and high-incidence areas, to enjoy cancer screening services free of charge and thus reduce the harm of cancers on the health of residents [11-14] (Table 1).

Local-level cancer screening projects are mainly screening projects launched for local special high-incidence cancers according to local conditions. For example, Tianjin launched the “Joint Screening for Common Cancers Project in Tianjin” in 2017, which targeted 4 local high-incidence cancers, that is, lung cancer, breast cancer, stomach cancer, and liver cancer [15]. In 2019, Hunan Province launched the “Oral Cancer Screening and Early Diagnosis and Treatment Project,” which targeted oral cancer, a local high-incidence cancer [16].

In addition, many charity organizations have implemented some cancer screening projects in China. For example, the “Healthy China - Maternal and Child Health Promotion Action” Central and Western China Cervical Cancer Screening Project, which was implemented jointly by China Population Welfare Foundation and the Maternal and Child Health Center of Chinese Center for Disease Control and Prevention, was launched in March 2023 [17]. The project is intended to explore a new mode of breast cancer and cervical cancer screening services in central and western China and help build the cervical cancer prevention and treatment capacity in central and western China. The “Healthcare Aid Project of Early Gastrointestinal Cancer Screening,” initiated jointly by Tencent Foundation, Guangdong Foundation for Poverty Alleviation, and Guangzhou United for Good Development Center, was launched on June 15, 2019 [18]. It provides early cancer screening and treatment assistance to poverty-stricken households and families in key poverty relief and development areas by offering medical AI technical support to some primary hospitals.

**Table 1:** The 4 national cancer screening programs in China.

Program	Start year	Prevention focus	Coverage
Cancer Screening Program in Rural Areas	2005	High-risk areas of upper gastrointestinal cancer	In 263 counties in 31 provinces (by 2020)
Cancer Screening Program in Huaihe River Area	2007	High-risk population of esophageal cancer, gastric cancer, and liver cancer	By 2019, a total of 32 counties in 4 provinces (Jiangsu, Shandong, Anhui, and Henan)
Cervical Cancer and Breast Cancer Screening Program for Women	2009	Women aged 35-64 years in rural target areas, cervical cancer, and breast cancer	In total, 87% (2644 counties) of counties in China for cervical cancer and 81% (2456 counties) of counties in China for breast cancer (by 2020)
Cancer Screening Program in Urban Areas	2012	Population risk factors survey, high-risk population assessment, cancer screening, and health economic evaluation were carried out for lung, breast, colorectal, esophagus, stomach, and liver	The initial 9 provinces in 2012 and 76 cities in 30 provinces by 2022

### Cancer Prevention and Control Working Modes

**Figure 2:**
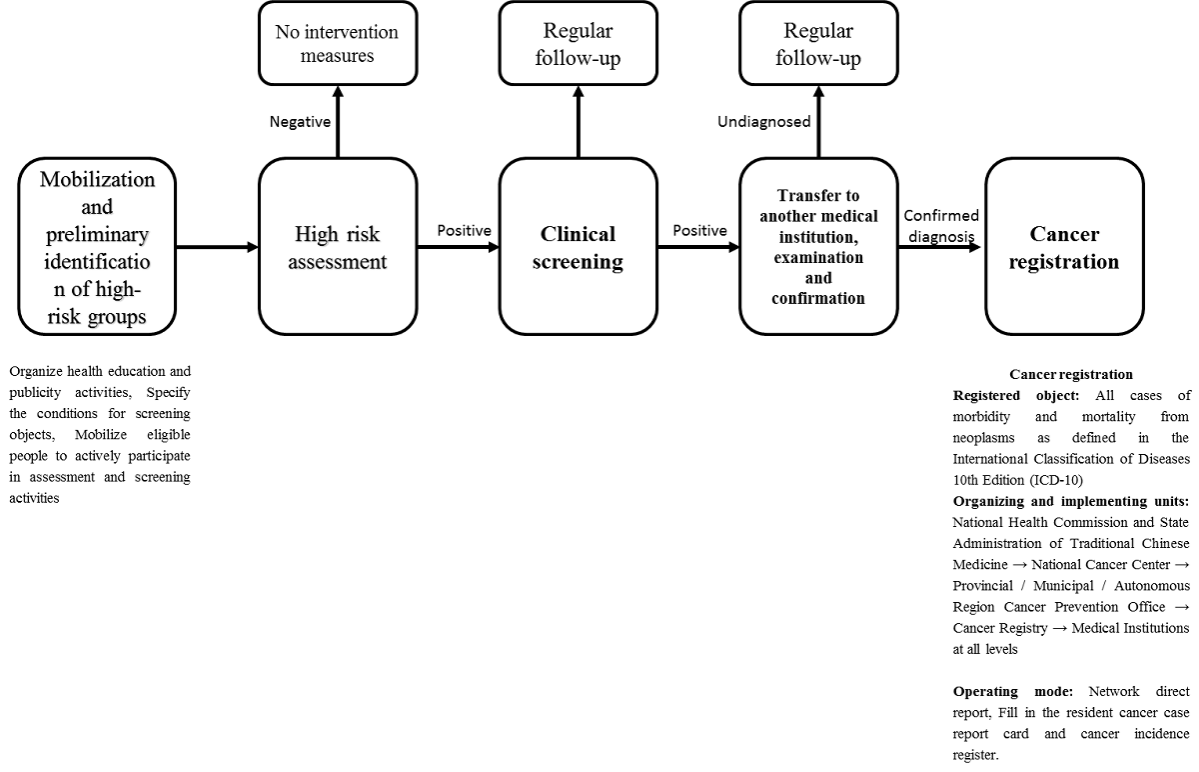
Cancer prevention and control working mode in China. ICD-10: International Classification of Diseases, 10th Edition.

The working modes and steps of cancer prevention and control projects in China are largely identical but with minor differences [19,20]. They are described roughly as follows (Figure 2).

- Mobilization and preliminary identification of high-risk groups: subdistricts, communities, and local health administration departments organize health education and publicity activities together; specify the conditions for screening service receivers; and mobilize eligible people, especially high-risk groups, to actively participate in assessment and screening activities.
- High-risk assessment: eligible screening service receivers who were willing to participate in a project fill in the questionnaire of risk factors under the guidance of appointed persons. The high-risk group assessment model and its background software are used. People who are assessed as high-risk groups are informed of receiving subsequent clinical cancer screening, while those who are assessed as non–high-risk groups are advised to maintain a healthy lifestyle and undergo regular physical examinations.
- Clinical screening: people who are assessed as high-risk groups go to the designated hospital to undergo relevant clinical cancer screening at the time specified. People who tested positive in a clinical screening will be assisted to go to another medical institution for re-examination and confirmation, and they will be followed up on regularly. Those who are tested negative in clinical screening will be followed up and intervened with regularly.
- Transfer to another medical institution, examination, and confirmation: suspected patients who test positive in clinical cancer screening will be recommended and assisted to move to a medical institution with cancer diagnosis and treatment capacities for physical examination and confirmation. Confirmed patients will be recommended to receive medical treatment and be registered. The project team will visit them regularly. Unconfirmed patients will be recommended to receive further consultation and be followed up regularly.
- Cancer registration: for confirmed cancer patients, per the *Measures for the Administration of Cancer Registration* and the cancer classification specified in the *International Classification of Diseases, 10th Edition*, all the medical institutions under the jurisdiction where the cancer registry is located should include the cancer information of patients in the *Residents’ Cancer Information Report Card* and *Cancer Incidence Register*. Further, they should then provide such information quarterly to the cancer registry under the jurisdiction through the information system or the registration report card. The cancer registry will file, code, verify, and analyze the data; make up the deficiency; remove repeated information; and then submit the data step-by-step to the provincial-level cancer prevention office and the National Cancer Center in the form of web-based direct reporting.

## Main Problems in Cancer Prevention and Control Projects at the Present Stage and Countermeasures

### Screening Coverage Is Relatively Limited

The cancer screening projects implemented in China currently mainly target common cancers and high-risk cancer areas or groups [21,22], covering limited cancers, regions, and groups. Take the urban cancer project as an example; currently, it covers 76 cities. Compared with the 293 prefecture-level cities in China, the project’s coverage is very limited. In addition, employees’ physical examinations—organized by some enterprises and public institutions where residents have spontaneous physical examinations and patient clinical examinations and in which cancer lesions are accidentally found—are also important ways to find cancers and precancerous lesions. However, since relevant physical examination, test, and diagnosis information has not been included in the current cancer prevention and control system, there is no way to track and manage such information in a standardized manner. For such problems, AI and other emerging information technologies may be taken into consideration to automatically capture massive health information, for example, in routine physical examination and hospital examination and diagnosis information, to accurately locate high-risk groups and patients and have them managed in a standardized manner. On this basis, cloud computing and intelligent algorithms may also be used to determine target high-risk residents for cancer screening targets based on massive population health data, to break the regional or population boundaries in traditional screening projects and make the projects cover all residents finally.

### Technological Level of Screening Services Is Not Balanced, and Screening Service Capacity and Technical Reserve Are Limited

The level of community and economic development varies in different places in China. Therefore, the medical service capacity, clinical screening hardware facilities, and medical personnel’s professional capacity in different regions are different. As a result, the level and quality of screening vary greatly [5]. In addition, organizers and managers fail to fully consider the screening service capabilities of screening service providers, for example, the technical level of clinical examinations and tests, the current workload, and workload expansion space. They only make a rough judgment and assign “indicator-based” tasks according to the level, functional positioning, and qualification of medical institutions. Such tasks may have an impact on daily business and work, increase the workload, and thus affect the quality of work [23,24]. How to make full use of cross-regional high-quality medical resources and technological means to assist areas with weak technology and workforce in improving their technical level of screening and service capacity through emerging information technologies, for example, telemedicine and AI-assisted imaging diagnosis, is a key issue that needs to be considered and put into practice.

### Efficiency of Defining High-Risk Groups of Cancers Needs to Be Increased

Efficiently and accurately locating high-risk groups of cancers is an important basis for accurate cancer prevention and control. Community-based questionnaire surveys are still the main channel used to obtain information on high-risk groups in cancer screening projects in China [25,26]. Since a huge population needs to be screened, such screening becomes an extra workload for community workers who are already busy with their routine work. Moreover, the screening is of huge human cost. Therefore, manual screening of high-risk groups by communities becomes a bottleneck restricting the efficient implementation of a whole screening project, and the accuracy and timeliness of collected information directly affect the efficiency and results of screening projects. At present, China has accumulated a certain scale of cancer monitoring data, cancer registration sample information, and cancer follow-up data [27], which lay a good foundation for the concentration of high-risk groups. On this basis, the use of big data technology, for example, data pipeline triggers during data ingestion or conditional logic in data analysis and visualization tools, to accurately identify high-risk groups and further locate high-risk individuals using e-survey can effectively reduce the workload of communities in high-risk group screening, improve the accuracy and effectiveness of defining high-risk groups, break through the bottleneck in current screening projects, and increase work output.

### Screening Service Receivers’ Insufficient Willingness to Enjoy Services Has an Impact on the Sustainability of Screening Projects

Residents’ awareness of the benefits that cancer screening can bring to themselves has an important impact on their participation in and compliance with screening projects. Studies [25,28-32] have found that most Chinese residents still lack awareness of cancer risk factors, knowledge of cancer prevention and control, and consciousness to prevent cancers. Especially, they know little about the importance of cancer screening and cancer prevention and treatment strategies. Due to the pain in the screening process for some cancers, people are reluctant to accept and comply with cancer screening. For these reasons, cancer screening services are underused, which has a direct impact on the overall implementation effect of screening projects and thus affects the long-term sustainability of screening projects. For the screening projects in progress currently, improving residents’ awareness of cancer risk factors, consciousness to prevent cancers, and willingness to participate in screening is the key to having more screening services used. In this demand context, efforts should be made to make use of new media and other publicity modes to push multilevel, all-round, and accurate information on cancer risk factors and screening for cancer prevention. This is to further enable more people to accept cancer screening on their own and lay a foundation for people to accept cancer screening stably and sustainably.

## Envisagement on Making Use of Emerging Information Technologies to Upgrade Cancer Prevention and Control

### Overview

Combined with the current situation of cancer prevention and control projects, we try to think about the application of emerging information technologies in various stages of cancer prevention and control. We describe these below (Tables 2 and 3).

**Table 2:** Current situation and potential solutions in various stages of cancer prevention and control.

Stage and current situation		Potential solutions
**Mobilization of high-risk groups and preliminary identification of high-risk groups**		
	Traditional publicity methods (for example, notification of doctors and neighborhood associations, poster campaign): the regional restrictions	Use new media platforms to shape more cancer prevention and control information dissemination channels Use big data technologies to collect and analyze the massive user interaction information on new media platforms and push personalized cancer prevention knowledge according to users’ screening demands to improve the cancer prevention and control information dissemination efficiency Bring the multiple roles of new media users to enhance the interaction and sense of substitution of publicity and intervention
	Traditional screening projects: the restrictions on regulations or populations	Expand data sources Use AI^a^ algorithm to analyze residents’ demands for cancer screening based on multisource data
**Cancer risk assessment**		
	Collect participants’ basic information, dietary habits, lifestyle and environment, psychology and emotion, and history and tumor cancer family history, through paper-based questionnaires	Replacement of paper questionnaires with online electronic questionnaires Use machine learning and biological information mining technologies to capture, summarize, develop, and use relevant data and information Identify key tumor cancer biomarkers related to the carcinogenetic process in combination with methods of epidemiology, statistics, and computational biology

^a^AI: artificial intelligence.

**Table 3:** Current situation and potential solutions in various stages of cancer prevention and control.

Stage	Current situation	Potential solutions
Clinical screening, physical examination, and confirmation	Clinicians interpret screening results The shortage of doctors or screening technologies in some areas	Help clinicians obtain image data and section information comprehensively and assist clinicians to interpret screening results with the help of the powerful biological data analysis ability and the deep machine learning ability of AI^a^-assisted diagnosis technologies Use the clinical auxiliary cancer decision system built based on AI and other technologies to assist doctors in making a diagnosis and decision on the people who tested positive in the clinical screening of screening projects
Cancer registration	Population-based cancer registration and hospital-based cancer registration	Rely on big data mining, AI, and information capture technologies to extract tumor cancer diagnosis, treatment, and other medical record information, as well as follow-up visit and risk factor information, in time and build a monitoring database with more accurate and detailed risk factors, incidence, and death information Integrating and complementing population-based and hospital-based cancer registration
Follow-up visits	Absence of a follow-up management system for screening projects	Build a follow-up visit management system for cancer prevention and control based on internet and AI-based machine learning technologies to realize the information interconnection with hospitals’ diagnosis and treatment systems and tumor cancer registration systems

^a^AI: artificial intelligence.

### Stage of Mobilization and Preliminary Identification of High-Risk Groups

At the stage of mobilization of high-risk groups, a new cancer screening publicity network ecosystem may be built based on various new media platforms by breaking through the regional restrictions on traditional publicity ways. New media platforms such as mobile communication tools (various apps), internet tools (blogs, microblogs [Sina, Tencent, and Sohu], and forums), and new television technology may be used to provide image, text, video, or audio content to publicize cancer risk factors, the identification of early cancer symptoms, and the important role of individuals in the process of cancer prevention to shape up more cancer prevention and control information dissemination channels. Big data technologies, for example, collaborative filtering, may be used to collect and analyze the massive user interaction information on new media platforms. After such information is optimized and integrated, personalized cancer prevention knowledge may be pushed according to users’ screening demands to improve the cancer prevention and control information dissemination efficiency. The multiple roles of new media users as information receivers, re-editors, and publishers may be brought into play to enhance the interaction and sense of substitution of publicity and intervention. Through around-the-clock and full-coverage cancer prevention and control information dissemination by new media, residents can improve their awareness of cancer prevention and more residents will take the initiative to accept cancer screening. Accordingly, more people will enjoy cancer screening services.

At the stage of preliminary identification of high-risk groups, AI technology may be used to extract big data related to residents’ health records, physical examination data, medical diagnosis information, and regional health data that are under standardized management. Cloud computing and intelligent algorithms may be used to determine residents’ demands for cancer screening. In this way, the restrictions on regulations or populations in traditional screening projects may be broken through and cancer prevention and control may cover more people and regions. AI algorithms were used to automatically extract the human health monitoring data from wearable devices, health consumption data in consumer payments, netizens’ demand data that are captured by internet search engines, users’ installation and use data that are collected by mobile apps, and residents’ physical examination data in the *Tsinghua City Health Index 2022*. The experience of the multisource social big data collection and extraction in the *Tsinghua City Health Index 2022* may be useful for the preliminary determination of high-risk groups in cancer screening projects. By analyzing residents’ demands for cancer screening based on multisource data, more people’s demands for cancer prevention may be covered.

### Stage of Cancer Risk Assessment

At the stage of risk assessment, machine learning and biological information mining technologies may be used to capture, summarize, develop, and use relevant data and information. In combination with methods of epidemiology, statistics, and computational biology, key cancer biomarkers related to the carcinogenetic process may be identified. At the same time, based on the resident health information accumulated by various interconnected and shared platforms, a high-risk cancer group identification model may be built to achieve accurate stratification and management of high-risk groups. Such technology has been applied both at home and abroad. For example, some researchers in the United States built a prostate cancer risk prediction model by using machine learning technologies and methods, and they found that the prediction result was good after verification [33]. Some researchers used biological information data mining technologies and they found specific signals enhancing the migration and invasiveness of nonsmall cell lung cancers and established a high-risk nonsmall cell lung cancer metastasis risk prediction model [34]. Chinese researchers built risk prediction models for the “onset” and “progress” of esophageal cancer. They found specific indicators affecting the accuracy of esophageal cancer progress risk prediction and achieved accurate and individualized esophageal cancer progress risk assessment [35].

Meanwhile, in the process of collecting data, for example, participants’ basic information, dietary habits, lifestyle and environment, psychology and emotion, history, and cancer family history, the screening projects’ online electronic questionnaire survey may be carried out. Thus, a variety of out-of-order data may be reasonably classified, simplified, well-organized, and standardized. Automatic sorting can help reduce workload sharply; reduce the screening workload of primary health institutions, that is, specific implementers of screening projects; decrease the service supply problems caused by insufficient service willingness resulting from excessive workload; and further lower the manpower cost for screening.

### Stage of Clinical Screening, Physical Examination, and Confirmation

At the stage of clinical screening, with the help of the powerful biological data analysis ability and deep machine learning ability of AI-assisted diagnosis technologies, the massive image and body fluid sample test data accumulated by cancer screening projects as preliminary diagnosis results of screening service receivers may be analyzed, and preliminary conclusions may be provided. These help clinicians obtain image data and section information comprehensively, assist clinicians in interpreting screening results, quickly screen diseases, and make a definite diagnosis to make up for the shortage of doctors or screening technologies in some areas and increase the efficiency and effect of screening. AI-based computer-aided diagnosis technologies have been widely used in many medical fields such as imaging and pathological diagnosis of cancers or precancerous lesions. For example, in terms of pulmonary nodule diagnosis, the AI-based accurate algorithm model can quickly detect nodules in a short time and predict benign and malignant nodules [36]. As such a model is combined with film reading by doctors of the imaging department, work efficiency and diagnostic accuracy have been greatly improved. For pathological sections of lung tissue, the AI model based on deep learning algorithms may be used to assist pathologists of Tongji Hospital of Tongji University and Shanghai Pulmonary Hospital to analyze stained pathological sections of lung lesion tissue [37]. The model has been connected with 2 modules, that is, the division of benign and malignant lesion areas and the classification of pathological subtypes, which can help users more sensitively detect malignant lesions and more accurately classify pathological subtypes. Good diagnostic results have been achieved. To rapidly assess living breast tissue, Loukas et al [38] have established a highly intelligent breast cancer image classification system based on AI-based computer-aided diagnosis technologies from 2 aspects of studying nuclear mechanism characteristics and breast cancer image distribution characteristics. The results showed that this system is entirely possible to replace artificial visual diagnosis.

At the stage of physical examination and diagnosis, the auxiliary consultation and auxiliary diagnosis modules of the clinical auxiliary cancer decision system built based on AI and other technologies may be used to assist doctors in multiple aspects to make a diagnosis and decision on the people who tested positive in the clinical screening of screening projects. These may be used to improve the efficiency of medical diagnosis, shorten the time of confirmation, enhance the diagnosis and treatment ability, alleviate the shortage of medical resources, provide support for standardizing personalized diagnosis and treatment schemes for patients, and realize early diagnosis and treatment of cancers. Similar systems have been built and applied in China. For example, the intelligent auxiliary multiple myeloma decision system built based on AI provides support for doctors’ clinical diagnosis and decision-making by mining and analyzing a large number of patients’ clinical diagnosis and treatment data and experts’ diagnosis and treatment experience [39]. The system has shown that the confirmation rate has increased, the time required for confirmation of patients has been significantly shortened, and the average length of stay and cost have also reduced sharply. Sichuan Cancer Hospital has designed and applied a clinical auxiliary cancer decision system according to its actual needs [40]. The system makes use of data mining technologies to automatically extract data related to patients’ medical records and related analysis indicators of medical record data and keeps improving the accuracy and execution efficiency of clinical decisions. In addition, a telemedicine platform for the Oncology Department has been established by using internet and data processing technologies. It is used to perform such functions as remote ultrasound examination, remote pathological examination, expert consultation, cancer diagnosis, and referral for cancer patients. It is also used to diagnose and treat cancers earlier, to provide convenient cancer diagnosis and treatment services for residents in remote and grassroots areas with a low diagnosis and treatment level and to alleviate the problem of uneven distribution of medical resources especially diagnosis and treatment resources of cancer departments in China.

### Stage of Cancer Registration

At the stage of cancer registration, efforts should be made to learn from the experience in Yanting in Sichuan Province, Shanghai, Jiaxing, and Ninghai in Zhejiang Province [41]. These are regions that fully rely on big data mining, AI, and information capture technologies to timely extract cancer diagnosis, treatment, and other medical record information, as well as follow-up visit and risk factor information, and build a monitoring database with more accurate and detailed risk factors, incidence, and death information. Separated population-based cancer registration should be integrated with hospital-based cancer registration, and both kinds of cancer registration should complement each other. A connection and information-sharing mechanism between cancer registration platforms and medical institutions in a wider range in the country should be established to further enable the functions of cross-regional information capture, integration, analysis, and release.

### Stage of Follow-Up Visits

At the stage of follow-up visits of screening projects, existing follow-up visit management experiences of patients with cancer [42,43] should be used for reference. Further, a follow-up visit management system for cancer prevention and control should be built based on internet and AI-based machine learning technologies to realize the information interconnection with hospitals’ diagnosis and treatment systems and cancer registration systems. With the help of the advantages of different platforms such as telecommunication networks, internet platforms, and mobile terminals, relevant information of follow-up recipients should be collected, supplemented, and improved constantly to automatically shape follow-up visit files. Based on the massive data collected, the next measures to be taken for follow-up recipients should be assessed. According to assessment results, relevant content should be intelligently pushed to follow-up recipients and medical personnel. Information of each follow-up visit should be automatically recorded and stored to realize such functions as screening service and process management, information collection, data visualization, and multiterminal support. It should also realize the automatic generation of treatment or follow-up visit arrays based on the pathological examination results of screening service receivers; realize interconnection with medical and health institutions in terms of admission, incidence, diagnosis, and ending information; and promote the complete and efficient management of cancer prevention and control efforts.

## Conclusion

Combined with the analysis of major problems in cancer prevention and control projects, this paper probes into how to apply emerging information technologies, for example, AI-based machine learning, data mining, and electronic information collection, to various stages of cancer prevention and control. Information technologies realize the integrated management of prevention and control processes, for example, mobilization and preliminary identification, high-risk assessment, clinical screening, clinical diagnosis and treatment, tracking and follow-up, and biological sample management of high-risk groups, and promote the efficient implementation of cancer prevention and control projects in China.

## Abbreviations

AI: artificial intelligence
